# Attention Deficit Hyperactivity Disorder‐Symptoms, Social Media Use Intensity, and Social Media Use Problems in Adolescents: Investigating Directionality

**DOI:** 10.1111/cdev.13334

**Published:** 2019-10-26

**Authors:** Maartje Boer, Gonneke Stevens, Catrin Finkenauer, Regina van den Eijnden

**Affiliations:** ^1^ Utrecht University

## Abstract

Cross‐sectional research shows that adolescents’ social media use (SMU) and attention deficit hyperactivity disorder (ADHD)‐symptoms are related, but it is unclear whether this relation is explained by SMU intensity or by addiction‐like SMU problems. Also, due to the lack of longitudinal studies, the direction of this relation remains unknown. This study aims to disentangle which type of SMU is related to ADHD‐symptoms, and in which direction, using a three‐wave longitudinal study among Dutch adolescents aged 11–15 years (*n* = 543). Findings suggest a unidirectional relation: SMU problems increased ADHD‐symptoms over time, SMU intensity did not. This implies that problematic use, rather than the intensity of use harmfully affects adolescents’ ADHD‐symptoms.

Social media use (SMU) such as the use of Instagram and Snapchat has increased over the last few years, especially among adolescents (Anderson & Jiang, 2018; Kloosterman & Van Beuningen, 2015). In 2018, 45% of the adolescents in the United States aged 13–17 reported being online almost constantly, while in 2015 this was 24% (Anderson & Jiang, 2018). Although SMU enables adolescents to stay involved with peers and facilitates engagement in online social activities (Kuss & Griffiths, 2011; Ryan & Xenos, 2011), scholars have raised concerns that SMU may increase symptoms of attention deficit hyperactivity disorder (ADHD) in youth (Cabral, 2011; Levine, Waite, & Bowman, 2007, 2012). However, it remains unclear which aspect of SMU would drive this association. To enhance our understanding of whether and how SMU and ADHD‐symptoms are related, this study distinguishes between *SMU intensity* and *SMU problems*. SMU intensity refers to the frequency of use, whereas SMU problems are characterized by addiction‐like behaviors, such as the displacement of other activities for SMU, or having conflicts with others due to their SMU (Griffiths, Kuss, & Demetrovics, 2014; Van den Eijnden, Lemmens, & Valkenburg, 2016). Although adolescents with SMU problems typically report high SMU intensity (Van den Eijnden, Koning, Doornwaard, Van Gurp, & Ter Bogt, 2018; Van den Eijnden et al., 2016), high SMU intensity does not necessarily impair important life domains to the same extent as SMU problems.

Cross‐sectional research showed that adolescents who reported high SMU intensity, also reported more ADHD‐symptoms (Barry, Sidoti, Briggs, Reiter, & Lindsey, 2017; Levine et al., 2007). Other studies found associations between SMU problems and ADHD‐symptoms (Andreassen et al., 2016; Mérelle, Kleiboer, Schotanus, Cluitmans, & Waardenburg, 2017; Van den Eijnden et al., 2016; Wu, Cheung, Ku, & Hung, 2013). These findings raise two questions. First, it remains unclear whether SMU intensity, SMU problems, or both relate to ADHD‐symptoms, because existing studies examined SMU intensity and SMU problems separately. Nevertheless, these two types of SMU are correlated (Van den Eijnden et al., 2018, 2016). Although theoretically both types of SMU can be related to ADHD‐symptoms, research has shown that SMU intensity and SMU problems can generate different outcomes over time (Van den Eijnden et al., 2018). Therefore, the first aim of this study is to explore whether the two types of SMU relate to ADHD‐symptoms. Second, given the cross‐sectional nature of previous research, the directions of the relations between social media behaviors and ADHD‐symptoms remain unknown. Using data from a longitudinal study, this study addresses these gaps in our knowledge.

Recently, scholars have cautioned against overpathologizing normative behaviors, questioning whether problematic internet‐related behaviors, as defined by substance addiction criteria, cause significant harm (Kardefelt‐Winther et al., 2017; Van Rooij et al., 2018). Yet, recent longitudinal research suggests that SMU problems impair life satisfaction over time, while SMU intensity does not (Van den Eijnden et al., 2018). This study extends this research by exploring whether SMU intensity and SMU problems independently, or in concert, increase ADHD‐symptoms.

## The Influence of ADHD‐Symptoms on SMU Intensity and SMU Problems

ADHD is characterized by three behavioral components: attention deficits, hyperactivity, and impulsivity. Adolescents with attention deficits often experience difficulties in completing tasks that require a long attention span, because they easily become distracted. Adolescents with hyperactive behavior typically show physical restlessness. Impulsive adolescents tend to have a strong preference for immediate rewards over delayed rewards, and often act without deliberate forethought (American Psychiatric Association, 2013).

Social media afford several features that may be particularly attractive to adolescents with ADHD‐symptoms. First, they can be used through smartphones at any time and at any place, and social media applications on smartphones actively notify users of incoming messages and updated content (Pielot, Church, & de Oliveira, 2014). Social media may therefore be tempting external distractors in daily life to which adolescents with ADHD‐symptoms are more sensitive than adolescents without symptoms (American Psychiatric Association, 2013). Second, social media allow adolescents to navigate through profiles quickly and to engage in multiple conversations at the same time, facilitating quick rewards to immediate informational and social needs. We therefore expected that *ADHD‐symptoms increase SMU intensity over time (H1).* Furthermore, ADHD‐symptoms constitute a risk factor for developing addictions, such as substance dependency (Cyders & Smith, 2009; Ohlmeier et al., 2008). Because SMU problems are characterized by addiction‐like behaviors, adolescents with ADHD‐symptoms may also be sensitive to developing SMU problems. We therefore expected that *ADHD‐symptoms increase SMU problems over time (H2).*

## The Influence of SMU Intensity and SMU Problems on ADHD‐Symptoms

Adolescents who intensively use social media may be accustomed to task‐switching between media activities and other (offline or online) activities (Karpinski, Kirschner, Ozer, Mellott, & Ochwo, 2013; Rosen, Whaling, Carrier, Cheever, & Rokkum, 2013). This may impair their ability to filter relevant from irrelevant information, which may, in turn, contribute to the development of attention deficits (Baumgartner, Van der Schuur, Lemmens, & Te Poel, 2017). Also, intensive social media users may become habituated to the entertainment provided by social media. As a result, they may perceive activities without media that require prolonged attention as unentertaining or boring, resulting in experiences of attention deficits (Nikkelen, Valkenburg, Huizinga, & Bushman, 2014). Furthermore, intensive SMU may disrupt sleep due to intensive exposure to bright screens (Van der Schuur, Baumgartner, & Sumter, 2018), which, in turn, could lead to more attention deficits or to impaired abilities to forego immediate impulses at daytime (Fallone, Acebo, Arnedt, Seiger, & Carskadon, 2001). We thus expected that *SMU intensity increases ADHD‐symptoms over time (H3).* Also, adolescents with SMU problems may experience attention deficits due to their preoccupation with social media. Their constant urge to go online may make them feel restless when they cannot immediately check and respond to incoming messages, for example, at school. We therefore expected that *SMU problems increase ADHD‐symptoms over time (H4).*

## Current Study

This study investigated the directionality of associations between ADHD‐symptoms and both SMU intensity and SMU problems, using three waves of longitudinal data on Dutch secondary‐school adolescents aged 11–15 years (Van den Eijnden, 2018). To address directionality, we applied the “random intercept cross‐lagged panel model” (RI‐CLPM; Hamaker, Kuiper, & Grasman, 2015). This novel modeling technique allows us to examine relations between social media behaviors and ADHD‐symptoms over time, while controlling for all possible confounding stable characteristics, such as personality traits. The technique draws on a multilevel approach by disentangling within‐ and between‐person variance, allowing for more accurate estimations of directionality (Hamaker et al., 2015).

## Method

### Sample

To examine our hypotheses, we used the first three waves of the Digital Youth‐project; a longitudinal study on online behaviors and mental health among secondary school students based on self‐report measures (Van den Eijnden et al., 2018). The study was conducted in February and March of 2015, 2016, and 2017, respectively. In the first wave, 543 adolescents from the first and second year of two secondary schools participated in the study. Both schools were based in the Netherlands: one school was located in medium‐sized city and the other was located in a large city. Participants were between 11 and 15 years old (*M*_age_ = 12.91, *SD*_age_ = .73). Of this sample, 293 adolescents (54%) participated in all three waves, 198 (36%) in two waves, and 52 (10%) in one wave. Nonresponse was mainly due to dropout of entire school classes and not due to individual selection, because teachers were absent, or because teachers were not able to schedule time for the completion of the survey. During the first wave, school year and gender were evenly distributed (51% first year students, 52% girls). Adolescents attending pre‐university education (48%) and adolescents with two Dutch parents (84%) were somewhat overrepresented compared to the composition of the Dutch adolescent population in the first 2 years of secondary school (26% and 73%, respectively; Statistics Netherlands, 2018).

Survey participation occurred through digital self‐completion during school hours and was voluntary and anonymous. Participants did not receive any incentives. Research‐assistants were present during assessments to assist when necessary. Participants were instructed that they were allowed to quit the survey at any time during assessment. Parents received information letters prior to survey participation, providing them the opportunity to refuse participation of their child. The study procedures were carried out in accordance with the Declaration of Helsinki and were approved by the board of ethics of the Faculty of Social Sciences at Utrecht‐University (FETC16‐076 Eijnden).

### Measures

#### SMU Intensity

Four items on the use of social network sites and instant messengers were used to measure SMU intensity (Van den Eijnden et al., 2018). Respondents were asked “How many times a *day* do you check social network sites?”, “How many times a *week* do you ‘like’ messages, photos, or movies from others on social network sites?”, “How many times a *week* do you send out a response to (or share) messages, photos, or movies from others on social network sites?”. Examples of social network sites were provided in the questionnaire (Facebook, Twitter, Instagram, Google+, or Pinterest). The fourth item referred to instant messenger use: “How many times a *day* do you send a message, photo or movie via your smartphone, via for example WhatsApp, Chat, SnapChat or SMS?”. Respondents answered on a 7‐point scale, where high values indicate high SMU intensity (0 = *less than once a day* or *week* and 7 = *more than 40 times a day* or *week*). Factor loadings of all items ranged between 0.68 and 0.82 across all three waves. Cronbach’s α values were .86 (T1), .85 (T2), and .84 (T3). The original scale consisted of six items. Items “How many times a week do you post a message, photo, or movie, on social network sites?” and “How many times a day do you check your smartphone on messages, photo’s, or videos, via for example WhatsApp, Chat, SnapChat or SMS?” were excluded due to having factor loadings below 0.5, and high intercorrelation (*r* = .70) with another item, respectively.

#### SMU Problems

The Social Media Disorder scale was used to measure SMU problems (Van den Eijnden et al., 2016). The scale includes nine items corresponding to the nine diagnostic criteria for Internet Gaming Disorder according to the Appendix of the *Diagnostic and Statistical Manual of Mental Disorders*, 5th ed. These criteria entail preoccupation, persistence, tolerance, withdrawal, displacement, escape, problems, deception, and conflict (Lemmens, Valkenburg, & Gentile, 2015), which are in line with criteria for substance dependence*.* Adolescents were asked “During the past year, have you (…),” followed by for example “regularly had no interest in hobbies or other activities because you would rather use social media?”, which refers to the criterion “displacement.” Respondents replied on a dichotomous scale (1 = *yes* and 0 = *no*). High values on the scale indicate a high level of SMU problems. Factor loadings ranged between 0.52 and 0.85 across all three waves. Prior validation research (Van den Eijnden et al., 2016) showed that the SMD‐scale had medium to large positive correlations with compulsive internet use and self‐declared social media addiction, confirming adequate convergent validity. The scale was also found to have small to moderate positive correlations with mental health problems and frequency of SMU, confirming satisfactory criterion validity. Given the dichotomous nature of the items, internal consistency was calculated using the ordinal alpha that is based on the tetrachoric correlation matrix (Gadermann, Guhn, Zumbo, & Columbia, 2012). Ordinal alpha values were .83 (T1), .90 (T2), and .89 (T3).

#### ADHD‐Symptoms

The ADHD‐Questionnaire was selected for use in this study, as it has been shown to be a reliable and valid measure of ADHD‐symptoms in adolescent populations (Scholte & Van der Ploeg, 1999). In order to gain insight into which ADHD‐symptoms related to social media behaviors, the three symptoms of ADHD were measured separately. *Attention deficits* was measured using nine items, for example “I avoid tasks that require prolonged effort.” Factor loadings ranged between 0.60 and 0.79; Cronbach’s α values were .89 (T1), .90 (T2), and .87 (T3). *Impulsivity* was indicated by six items, such as “I find it difficult to wait for my turn.” Factor loadings ranged between 0.55 and 0.77; Cronbach’s α values were .79 (T1), .83 (T2), and .81 (T3). Six items were used to measure *hyperactivity*, for example “I feel restless.” Factor loadings ranged between 0.47 and 0.85; Cronbach’s α values were .85 (T1), .88 (T2), and .82 (T3). Respondents replied on 5‐point response scales, where high values indicate higher levels of ADHD‐symptoms (1 = *never* and 5 = *very often*).

### Measurement Invariance Over Time

To draw conclusions on effects over time, identical constructs should be measured across all three waves. Therefore, *measurement invariance* analyses were conducted prior to the analyses, using Mplus 8.1 (Muthén & Muthén, 2017). For each measure, this was done by means of multigroup confirmatory factor analysis (CFA) on the data structured in long format (*n* = 1,629), where groups were indicated by waves. Measurement invariance was imposed by constraining the loadings and intercepts of the items to be equal across all waves, after which model fit was evaluated. For SMU problems, thresholds instead of intercepts were constrained to be equal, because this scale consists of binary items. Measurement invariance analyses for SMU intensity, attention deficits, impulsivity, and hyperactivity were carried out using Maximum Likelihood estimation with robust standard errors (MLR), which corrects for the somewhat skew distributions of these measures. For SMU problems, weighted least square means and variance adjusted (WLSMV)‐estimation was used, which is recommended for categorical items (Muthén & Muthén, 2017). For each multigroup CFA, overall model fit was evaluated using the comparative fit index (CFI; > .9 = acceptable; > .95 = excellent), Tucker–Lewis index (TLI; > .9 = acceptable; > .95 = excellent), and root mean square error of approximation (RMSEA; < .08 = acceptable; < .05 = excellent; Van de Schoot, Lugtig, & Hox, 2012). We subsequently evaluated whether removing the equality constraints on the loadings and intercepts/thresholds would significantly improve model fit based on change in CFI (increase of ≥ .010) and RMSEA (decrease of ≥ .015; Chen, 2007). In measurement invariance analyses, evaluation of model fit using ΔCFI and ΔRMSEA are preferred over chi‐square‐difference tests, because the latter is sensitive to large sample sizes (Chen, 2007; Cheung & Rensvold, 2002).

Table 1 shows that when measurement invariance over time was imposed, the overall model fits of the multigroup CFA models for SMU intensity, SMU problems, attention deficits, and impulsivity were all acceptable to excellent. Model fits did not significantly improve when equality constraints on the item loadings and intercepts or thresholds were released. This means that measurement invariance was established for these four measures, and that we can make meaningful conclusions about their longitudinal relations (Van de Schoot et al., 2012). Overall model fit for hyperactivity was relatively low (CFI = .874, TLI = .879, and RMSEA = .122), and measurement invariance was not established (ΔCFI = .019). However, additional analyses (results not shown) showed that measurement invariance was only related to the intercepts of two items from the hyperactivity‐scale. Hyperactivity was thereby sufficiently invariant over time for the purposes of our analyses (Van de Schoot et al., 2012).

**Table 1:** Measurement Invariance Analysis: Multigroup CFA (*n* = 1,629)

	Overall model fit constrained model^a^			Change in model fit^b^	
	CFI	TLI	RMSEA	ΔCFI	ΔRMSEA
SMU intensity	.989	.989	.047	.009	−.010
SMU problems	.963	.957	.034	−.007	.006
Attention deficits	.932	.935	.073	.009	.007
Impulsivity	.987	.987	.031	.004	.002
Hyperactivity	.874	.879	.122	.019	.026

Note

SMU = social media use; CFA = confirmatory factor analysis; CFI = comparative fit index; TLI = Tucker–Lewis index; RMSEA = root mean square error of approximation.

a Multigroup CFA model where item loadings and intercepts/thresholds were constrained to be equal over time.

b Compared to multigroup CFA model where item loadings and intercepts/thresholds were free to vary over time.

### Generating Factor Scores

Modeling the RI‐CLPM using latent variables for our measures was not feasible, given the complexity of our model related to the large number of latent variables. We therefore considered using the sum‐scores of the observed items, which is the most common practice in applications of the RI‐CLPM (Hamaker et al., 2015). However, the distribution of the sum‐score of SMU problems is heavily skewed (Van den Eijnden et al., 2016), which often leads to biased results in statistical analyses (Hox, Maas, & Brinkhuis, 2010). Moreover, sum‐scores do not consider that items have different contributions to their latent measure, as reflected by their different factor loadings, which may lead to inaccurate representations of latent measures (Distefano, Zhu, & Mîndrilă, 2009). We addressed these shortcomings by using *factor scores* instead of sum‐scores, which are imputed values that reflect plausible values of latent measures based on the CFA‐model (Distefano et al., 2009).

Factor scores were computed using Mplus 8.1. For all five measures separately, CFA models with three latent measures were specified, referring to the three repeated measures in wide format (*n* = 543). In these models, measurement invariance over time was imposed, and means of the latent measures were freely estimated. Factor scores according to these CFA models were computed and saved. The saved factor scores were subsequently used as observed variables for the RI‐CLPM. Factor scores of SMU intensity, attention deficits, impulsivity, and hyperactivity were computed using MLR‐estimation. WLSMV‐estimation was used to compute factor scores of SMU problems. Factor scores for participating as well as dropout cases were calculated based on all available data on previous wave(s). For example, for respondents that dropped out in the second wave, regression methods were used to estimate factor scores at the second wave using the respondents’ available scores at the first and third wave and the estimated model parameters (Muthén, 2004). Therefore, all 543 participants were retained in the analysis. Table 2 shows the descriptive statistics of the factor scores for all five measures in long format (*n* = 1,629).

**Table 2:** Descriptive Statistics, Factor Scores (*n* = 1,629)

	*M* [95% CI]	*SD*	Minimum	Maximum
SMU intensity	.22 [.16, .28]	1.22	−2.62	2.53
SMU problems	.14 [.12, .17]	0.49	−0.44	2.14
Attention deficits	.12 [.09, .16]	0.76	−1.37	2.95
Impulsivity	.01 [−.01, .04]	0.54	−0.87	2.52
Hyperactivity	02 [−.02, .06]	0.81	−1.17	2.89

Note

SMU = social media use.

Differences between participating and dropout participants were analyzed by predicting dropout in T2 and T3 with the computed factor scores of previous wave(s). Multivariate logistic regression (results not shown) showed that adolescents who reported high SMU intensity in T1 were more likely to dropout in T3 (OR = 1.34, *p* < .05), although this only explained a small proportion of the variance in T3 dropout (Nagelkerke *R* ^2^ = .010). SMU problems, attention deficits, impulsivity, and hyperactivity were not related to dropout in any of the waves.

### Modeling Strategy

Directionality can be established by examining whether changes in ADHD‐symptoms induce changes in social media behaviors, and vice versa, which refers to a dynamic process that takes place within adolescents. To study these dynamics within adolescents, between‐person variance should be separated from the within‐person variance, because time‐invariant traits on the between‐person level may confound within‐person dynamics. The RI‐CLPM partials out all possible confounding time‐invariant traits by adding a RI for each measure, which captures the stability of the respective measure at the between‐person level. As a result, cross‐lagged relations in the RI‐CLPM solely reflect within‐person dynamics that are not confounded by time‐invariant traits at the between‐person level (Hamaker et al., 2015), such as stable individual differences in temperament.

**Figure 1:**
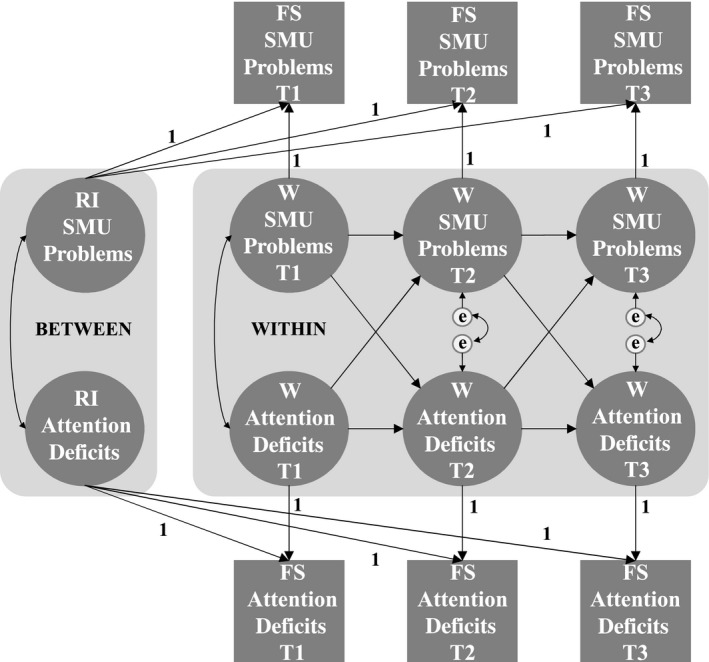
Two‐variable random intercept (RI) cross‐lagged panel model. Squares represent the computed factor scores (FS). Circles represent RIs and within‐person (W) values of the respective factor scores. On the within‐person level, cross‐lagged paths are denoted by the diagonal arrows, auto‐regressive paths by the horizontal arrows, and within‐wave (residual) correlations by the double‐ended arrows. Auto‐regressive paths, cross‐lagged paths, and within‐wave (residual) correlations were estimated freely. On the between‐person level, RIs were correlated. In the final analysis, this model has been extended with social media use (SMU) intensity, impulsivity, and hyperactivity.

After measurement invariance was established and factor scores were generated, the RI‐CLPM was fitted following the modeling strategy of Hamaker (2018) using Mplus 8.1 with MLR‐estimation. A two‐variable RI‐CLPM is illustrated in Figure 1. In this study this model was extended to a five‐variable RI‐CLPM, including SMU intensity, SMU problems, attention deficits, impulsivity, and hyperactivity (see Figure S1). The *between‐person part* of the RI‐CLPM is denoted by the RIs. RIs are latent variables that are extracted from the computed factor scores that reflect the same construct over time with loadings fixed to one. Each RI represents the person‐specific time‐invariant stability of the measure. Correlations between all RIs were specified. Positive correlations between the RIs indicate, for example, that adolescents with high averages in attention deficits also report high averages in SMU problems. The *within‐person part* of the RI‐CLPM is denoted by within‐person values, which are additional latent variables that are extracted from their respective computed factor scores, again with loadings fixed to one. Residual variances of the computed factor scores were constrained to zero. The within‐person values denote the adolescent’s deviations from their expected score. The expected score at *T_x_* consists of the grand mean of the respective wave and the adolescent’s RI. Cross‐lagged paths, auto‐regressive paths, and within‐wave (residual) correlations were specified between the within‐person values (Figure 1). Positive cross‐lagged paths indicate, for example, that adolescents whose attention deficits at *T_x_* increased relative to their expected score, also reported increased SMU problems relative to their expected score at *T_x_*_+1_. By including auto‐regressive paths, the model controls for preceding increases or decreases (e.g., SMU problems at *T_x_* on SMU problems at *T_x_*_+1_). By including within‐wave (residual) correlations, the model also controls for increases or decreases that occurred simultaneously within the same year (e.g., attention deficits at *T_x_* with SMU problems at *T_x_*). In addition, all cross‐lagged paths, auto‐regressive paths, within‐wave (residual) correlations, and means were unconstrained over time. Results of the RI‐CLPM were standardized (STD_YX_) to facilitate interpretation of effect sizes.

Monte Carlo simulations in Mplus 8.1 were carried out to determine statistical power to reject the null hypothesis of no effect (Muthén & Muthén, 2002). Power analyses were carried out using 1,000 simulated samples, a sample size of *n* = 543, and a Type I error rate of 0.05. The power analyses were based on our RI‐CLPM including free estimation of all cross‐lagged effects, auto‐regressive effects, and all (residual) correlations. For detection of moderate effects (β = .3), power ranged between 0.94 and 1 for all estimates. For detection of small effects (β = .2) power ranged between 0.68 and 0.94 for all estimates. We could not derive the minimum relevant effect size from the literature, because no longitudinal studies specifically focusing on social media behaviors and ADHD‐symptoms exist. Cross‐sectional studies examining the relation between (problematic) SMU and ADHD‐symptoms using multivariate models showed small‐to‐medium effect sizes with β = .24 on average (Andreassen et al., 2016; Barry et al., 2017; Levine et al., 2007; Mérelle et al., 2017; Wu et al., 2013)*.* For this effect size, power ranged between 0.80 and 0.99 for all estimates in our model. Thus, the analyses showed that our sample size of *n* = 543 is able to detect effect sizes corresponding to previous cross‐sectional studies.

## Results

### Preliminary Results

Prior to the main analysis, preliminary analyses were conducted on the data in long format (*n = *1,629) to study the intraclass correlations (ICCs) of our measures (i.e., the computed factor scores). ICCs express the proportion of variance that is explained on the between‐person level relative to the total variance, which provides insight into the stability of our measures over time. Table 3 shows that the majority of the measures in our study varied mainly between adolescents, especially for SMU problems (89.9%). This means that most of our measures were relatively stable over time. However, a substantial part of the variance of our measures was related to changes within adolescents over time (10.1%–28.5%).

**Table 3:** Preliminary Results, Standardized (*n* = 1,629)

	SMU intensity			SMU problems			Attention deficits			Impulsivity			Hyperactivity		
	β	*SE*	*p*	β	*SE*	*p*	β	*SE*	*p*	β	*SE*	*p*	β	*SE*	***p***
Wave 2^a^	.39	.06	< .001	.84	.05	< .001	.27	.06	< .001	−.05	.07	.476	.09	.06	.117
Wave 3^a^	.84	.06	< .001	.35	.06	< .001	.67	.07	< .001	.20	.06	.001	.09	.07	.163
Girls^b^	.42	.09	< .001	.22	.08	.007	−.07	.09	.445	−.18	.09	.046	−.02	.09	.842
Prevocational education^c^	.26	.09	.003	.45	.09	< .001	.25	.10	.010	.28	.09	.003	.26	.10	.007
Native ethnic background^d^	.25	.12	.034	−.03	.012	.831	.19	.13	.150	.02	.14	.871	.26	.11	.021
ICC^e^	.803			.899			.715			.771			.756		

Note

Results represent multilevel multiple regression results estimated with Maximum Likelihood with Robust standard errors. Observations (*n* = 1,629) were nested in individuals (*n* = 543). Waves were specified on the within‐person level; girls, educational level, and ethnic background were specified on the between‐person level. All independent covariates are binary, and therefore all coefficients were standardized based on STD_Y_‐standardization. SMU = social media use; ICC = intraclass correlation.

a Ref. = wave 1.

b Ref. = boys.

c Ref. = intermediate/pre‐university.

d Ref. = immigrant background.

e ICC = variance between/(variance within + variance between).

We also studied how our measures developed over time and whether the measures were related with demographic characteristics by means of multilevel multiple regression (*n = *1,629). Table 3 shows that on average, SMU intensity, SMU problems, and attention deficits increased in the second and the third wave relative to the first wave. Impulsivity only increased in the third wave relative to the first wave. Hyperactivity did not increase over time, although the ICC indicated that hyperactivity varied across waves. Girls reported higher SMU intensity and more SMU problems than boys. Girls also experienced less impulsivity than boys. Pre‐vocational educated adolescents reported higher SMU intensity, more SMU problems, and more ADHD‐symptoms than intermediate or pre‐university educated adolescents. Adolescents with two Dutch parents reported more SMU intensity and more hyperactivity than adolescents with at least one parent from another country. These observed mean differences in factor scores do not affect our longitudinal results, because the RI‐CLPM controls for all possible time‐invariant confounders, which makes adding between‐person characteristics as covariates redundant (Hamaker et al., 2015).

### ADHD‐Symptoms, SMU Intensity, and SMU Problems

The overall model fit of the RI‐CLPM was good (CFI = .998; TLI = .984; RMSEA = .042; χ^2^(10) = 19.472, *p* = .035, results not shown). Table 4 shows the correlations between the RIs. Adolescents with high averages of SMU intensity and with high averages of SMU problems also reported high averages in attention deficits, impulsivity, and hyperactivity (correlations varying from *r = *.23–.29, *p* = < .001–.032). Adolescents who reported high averages of SMU intensity also reported high averages of SMU problems (*r = *.40, *p* < .001).

**Table 4:** RI‐CLPM, Between‐Person Correlations (*n* = 543)

	SMU intensity			SMU problems			Attention deficit			Impulsivity		
	*r*	*SE*	*p*	*r*	*SE*	*p*	*r*	*SE*	*p*	*r*	*SE*	*p*
SMU intensity	1.00											
SMU problems	.40	.08	< .001	1.00								
Attention deficit	.23	.06	< .001	.24	.11	.032	1.00					
Impulsivity	.23	.06	< .001	.23	.11	.031	.67	.05	< .001	1.00		
Hyperactivity	.29	.06	< .001	.29	.10	.003	.63	.07	< .001	.64	.05	< .001

Note

SMU = social media use; RI‐CLPM = random intercept cross‐lagged panel model.

Table 5 depicts the auto‐regressive and cross‐lagged effects at the within‐person level. Results for Hypotheses 1 and 2 are denoted by the light gray cells in the table and are all nonsignificant. Specifically, adolescents whose ADHD‐symptoms increased did not report increases in SMU intensity 1 year later, nor did they report increased SMU problems 1 year later. These findings refute Hypotheses 1 and 2.

**Table 5:** RI‐CLPM, Standardized Within‐Person Cross‐Lagged Effects (*n* = 543)

(T2 →)	SMU intensity			SMU problems			Attention deficit			Impulsivity			Hyperactivity		
	β	*SE*	*p*	β	*SE*	*p*	β	*SE*	*p*	β	*SE*	*p*	β	*SE*	*p*
(T1 ↓)															
SMU intensity	.10	.15	.506	.02	.05	.758	.05	.08	.508	.03	.10	.739	.10	.08	.221
SMU problems	.31	.21	.140	.79	.04	< .001	.31	.11	.004	.19	.13	.150	.07	.09	.409
Attention deficit	−.03	.18	.857	−.04	.05	.421	.42	.12	.001	.05	.13	.721	−.08	.09	.391
Impulsivity	−.06	.18	.735	.13	.08	.090	−.08	.17	.623	.07	.17	.671	.03	.14	.857
Hyperactivity	.14	.16	.380	−.04	.04	.413	−.06	.12	.611	.19	.11	.094	.53	.10	< .001

(T3 →)	SMU intensity			SMU problems			Attention deficit			Impulsivity			Hyperactivity		
	β	*SE*	*p*	β	*SE*	*p*	β	*SE*	*p*	β	*SE*	*p*	β	*SE*	*p*
(T2 ↓)															
SMU intensity	.29	.29	.311	−.05	.06	.447	−.13	.20	.511	−.08	.16	.602	−.04	.19	.831
SMU problems	.33	.24	.163	.99	.04	< .001	.50	.21	.016	.51	.14	< .001	.28	.20	.158
Attention deficit	−.26	.22	.248	−.01	.05	.910	.19	.24	.431	−.19	.18	.281	−.26	.22	.241
Impulsivity	.13	.17	.450	−.05	.04	.294	.19	.15	.191	.38	.14	.008	.14	.16	.382
Hyperactivity	.08	.20	.678	.00	.05	.999	−.26	.16	.112	.04	.13	.763	.34	.18	.054

Note

Light grey cells depict results for Hypotheses 1 and 2; Dark grey cells depict results for Hypotheses 3 and 4. All coefficients were STD_yx_‐standardized. SMU = social media use; RI‐CLPM = random intercept cross‐lagged panel model.

The dark gray cells in Table 5 depict results for Hypotheses 3 and 4. Adolescents whose SMU intensity increased did not report increases in ADHD‐symptoms 1 year later, because we did not find cross‐lagged effects between SMU intensity and ADHD‐symptoms. This finding fails to support Hypothesis 3. However, adolescents whose SMU problems increased, also experienced increased attention deficits 1 year later, both from T1 to T2 (β = .31, *p* = .004) and from T2 to T3 (β = .50, *p* = .016). Comparison of unstandardized effect sizes using a Wald‐test indicated that the strength of these found relations were not significantly different (χ^2^(1) = 0.03, *p = *.870). Also, adolescents who experienced increased SMU problems at T2, reported increased impulsivity at T3 (β = .51, *p* < .001). The strength of this relation was equal to the relation between SMU problems at T2 and attention deficit at T3 (χ^2^(1) = 0.41, *p = *.522). However, increased SMU problems at T1 did not increase impulsivity at T2. We also did not find that increased SMU problems increased hyperactivity over time. Considering these results, Hypothesis 4 is partially confirmed.

### Additional Findings

Although adolescents who reported high SMU intensity also reported more SMU problems at the between‐person level (Table 4), the results in Table 5 show that on the within‐person level, adolescents whose SMU intensity increased did not report increased SMU problems 1 year later. Neither did adolescents whose SMU problems increased report increased SMU intensity 1 year later. In addition, adolescents whose SMU problems increased also reported increased SMU problems 1 year later, across all waves with relatively large effect sizes (from T1 to T2 β = .79, *p* < .001; from T2 to T3 β = .99, *p* < .001). This suggests that increased SMU problems were persistent over time. Such a pattern was not observed regarding SMU intensity. Also, adolescents whose attention deficits increased at T1 reported increased attention deficits at T2 (β = .42, *p* = .001), increased hyperactivity at T1 was associated with increased hyperactivity at T2 (β = .53, *p* < .001), and increased impulsivity at T2 was associated with increased impulsivity at T3 (β = .38, *p* = .008).

In addition, all measures at the within‐person level were positively correlated within T1 (Table 6). This means that during this wave, increases in SMU intensity, SMU problems, attention deficits, impulsivity, and hyperactivity occurred simultaneously. These associations were also found in T2, with the exception that increases in SMU intensity were not correlated with increases in attention deficits or impulsivity. During T3, increases in SMU intensity were not associated with increases in ADHD‐symptoms in the same wave, but SMU problems increased simultaneously with impulsivity.

**Table 6:** RI‐CLPM, (Residual) Correlations Within Waves (*n* = 543)

(T1 →)	SMU intensity			SMU problems			Attention deficit			Impulsivity		
Correlations (T1 ↓)	*r*	*SE*	*p*	*r*	*SE*	*p*	*r*	*SE*	*p*	*r*	*SE*	*p*
SMU intensity	1.00											
SMU problems	.38	.10	< .001	1.00								
Attention deficit	.26	.08	.001	.42	.09	< .001	1.00					
Impulsivity	.40	.08	< .001	.55	.10	< .001	.69	.06	< .001	1.00		
Hyperactivity	.40	.07	< .001	.31	.10	.002	.50	.08	< .001	.63	.06	< .001

(T2 →)	SMU intensity			SMU problems			Attention deficit			Impulsivity		
Residual correlations (T2 ↓)	*r*	*SE*	*p*	*r*	*SE*	*p*	*r*	*SE*	*p*	*r*	*SE*	*p*
SMU intensity	1.00											
SMU problems	.43	.10	< .001	1.00								
Attention deficit	.16	.15	.295	.44	.07	< .001	1.00					
Impulsivity	.25	.15	.089	.46	.07	< .001	.64	.07	< .001	1.00		
Hyperactivity	.39	.14	.007	.40	.05	< .001	.57	.06	< .001	.53	.07	< .001

(T3 →)	SMU intensity			SMU problems			Attention deficit			Impulsivity		
Residual correlations (T3 ↓)	*r*	*SE*	*p*	*r*	*SE*	*p*	*r*	*SE*	*p*	*r*	*SE*	*p*
SMU intensity	1.00											
SMU problems	.06	.10	.575	1.00								
Attention deficit	−.11	.19	.574	.12	.11	.256	1.00					
Impulsivity	−.07	.13	.586	.17	.07	.022	.51	.08	< .001	1.00		
Hyperactivity	−.11	.16	.491	.02	.09	.793	.31	.15	.048	.34	.11	< .001

Note

All coefficients were STD_yx_‐standardized. SMU = social media use; RI‐CLPM = random intercept cross‐lagged panel model.

## Discussion

This study investigated the direction of the relation between ADHD‐symptoms and both SMU intensity and SMU problems among adolescents, using longitudinal data. Over time, SMU problems, but not SMU intensity, increased ADHD‐symptoms. Specifically, we consistently found that adolescents, whose SMU problems increased, also experienced increased attention deficits 1 year later. Adolescents’ increased SMU problems at T2 also increased their impulsivity at T3. Yet, adolescents whose ADHD‐symptoms increased neither reported increased SMU intensity 1 year later nor did they report increased SMU problems 1 year later.

The finding that adolescents’ SMU problems increased ADHD‐symptoms 1 year later, while SMU intensity did not, provides several insights. First, it suggests that the impact of SMU on ADHD‐symptoms was not driven by the frequency of use, but rather by the addiction‐like aspect of problematic use, such as constant urge to go online or the inability to control SMU. Second, it supports the idea that SMU problems—as defined by substance dependence criteria—have harmful implications, which has been contested in scholarly debates (Kardefelt‐Winther et al., 2017; Van Rooij et al., 2018). Previous longitudinal analyses showed that SMU problems, but not SMU intensity, diminished life satisfaction over time (Van den Eijnden et al., 2018). Extending these findings, this study suggests that SMU problems also increase ADHD‐symptoms, whereas SMU intensity does not. Third, the finding that SMU intensity did not increase ADHD‐symptoms over time suggests that the intensive use of social media may be a normative behavior that is integrated into adolescents’ daily lives rather than a problematic behavior. The additional finding that increased SMU intensity did not precede increased SMU problems 1 year later supports this idea.

The longitudinal association between SMU problems and ADHD‐symptoms was most pronounced from T2 (2016) to T3 (2017), when increases in SMU problems not only predicted increases in attention deficit, but also increases in impulsivity. This may be because social media platforms became more advanced during this period. For example, Instagram—a social network site for sharing photos through a personal profile—was extended in 2016 with the possibility to share “Stories,” which is a series of photos or videos that disappear after 24 hr. Also, Snapchat—a popular instant messenger for sharing photos that disappear after 10 s—provided extra incentives for their users from 2016 onwards to use it more intensively, for instance through the launch of “Snapstreaks,” which indicate the number of consecutive days users exchanged photos with particular friends (Werning, 2017). The new affordances may have made social media even more attractive to adolescents and made them harder to resist. These changes may tax adolescents’ self‐control more heavily, in turn increasing ADHD‐symptoms.

We did not find support for our proposition that adolescents with more ADHD‐symptoms would be particularly attracted to the features of social media. Although adolescents with more ADHD‐symptoms are sensitive to developing addiction‐like behaviors, such as substance dependence (e.g., Ohlmeier et al., 2008), we did not observe this sensitivity for the development of SMU problems. Social media are possibly more salient in the daily lives of adolescents than substances. Therefore, SMU problems may be different in their etiology from substance dependence. Alternatively, our study design and method might have prevented us from observing an effect of ADHD‐symptoms on social media behaviors. Specifically, ADHD‐symptoms may have affected social media behaviors at a younger age, not included in our study. Furthermore, the measurement occasions were a year apart, while behaviors may influence each other within a shorter time interval. Also, adolescents’ initial level of ADHD‐symptoms at the between‐person level (e.g., genetically determined) may have influenced changes in social media behaviors. The within‐person oriented study design of the RI‐CLPM does not eliminate the possibility that stable levels of ADHD‐symptoms at the between‐person level affected social media behaviors over time.

An additional finding was that adolescents who experienced increased SMU problems were likely to experience increased SMU problems 1 year later as well, with high effect sizes. Scholars have questioned whether SMU problems, indicated by symptoms of addiction, reflect actual behavioral addiction symptoms. They have put forward that the behavior should lead to significant impairment, and that it should persist over time (Kardefelt‐Winther et al., 2017). The finding that SMU problems have harmful implications over time, and that they are highly likely to persist over time, supports the suggestion that SMU problems as defined in this study reflect behavioral addiction symptoms.

### Strengths, Limitations and Future Directions

This study has important strengths related to the research design. By disentangling within‐ and between‐person effects, we controlled for all possible confounding time‐invariant traits. The findings of this study are therefore an important first step in answering the question of directionality. By distinguishing two types of social media behaviors and three symptoms of ADHD, we gained a better understanding of the relation between specific elements of both social media behaviors and of ADHD‐symptoms. However, the self‐report measures used in this study may deviate from observed ADHD‐ and social media behaviors (Orben & Przybylski, 2019). Also, due to the use of long time‐intervals, potential relations between daily fluctuations in ADHD‐symptoms and social media behaviors could not be observed. Additionally, time‐varying covariates that are not included in the study may have contributed to the found associations. For example, age may have played a role in the found relations over time, because during adolescence SMU intensity typically increases with age (Boer & Van den Eijnden, 2018). Furthermore, the convenience sample and the somewhat overrepresented native and pre‐university adolescents relative to the general adolescent population in the Netherlands limit the generalizability of our findings.

Taking these limitations into account, more longitudinal research on social media behaviors and ADHD‐symptoms using more waves and shorter time‐intervals, with larger and more representative samples is desired to confirm the unidirectional conclusion of this study. More specifically, future research using smartphone applications that measure time spent on (specific) social media in combination with momentary assessments of ADHD‐symptoms may provide more objective (and specific) insights into the relation between SMU intensity and ADHD‐symptoms over time (Orben & Przybylski, 2019). Another promising direction for future research would be the investigation of the longitudinal relations between social media behaviors and ADHD‐symptoms for different subgroups separately, because particular groups (e.g., girls, low‐educated) may be more susceptible to media effects (Valkenburg & Peter, 2013).

### Conclusion

To conclude, findings from this longitudinal study suggest that SMU problems increase ADHD‐symptoms among adolescents, but SMU intensity does not. Moreover, our findings indicate that the relation was unidirectional, because the reverse pattern was not observed. This study extends current knowledge obtained from cross‐sectional research, and highlights the importance of distinguishing SMU problems from SMU intensity in understanding the relation between ADHD‐symptoms and social media behaviors. While SMU intensity may not be harmful, SMU problems need to be recognized as harmful to adolescent mental health.

## Supporting information


**Figure S1. **Simplified Illustration of the Five‐Variable Random Intercept Cross‐Lagged Panel Model
