# Supplementary material for: Attention Deficit Hyperactivity Disorder‐Symptoms, Social Media Use Intensity, and Social Media Use Problems in Adolescents: Investigating Directionality
Source: Child Dev. 2019 Oct 26;91(4):e853–65. doi: 10.1111/cdev.13334 (PMC7497191; doi:10.1111/cdev.13334)
Supplement: Supplementary file 1 — Figure S1. Simplified Illustration of the Five‐Variable Random Intercept Cross‐Lagged Panel Model [file CDEV-91-e853-s001.pdf]

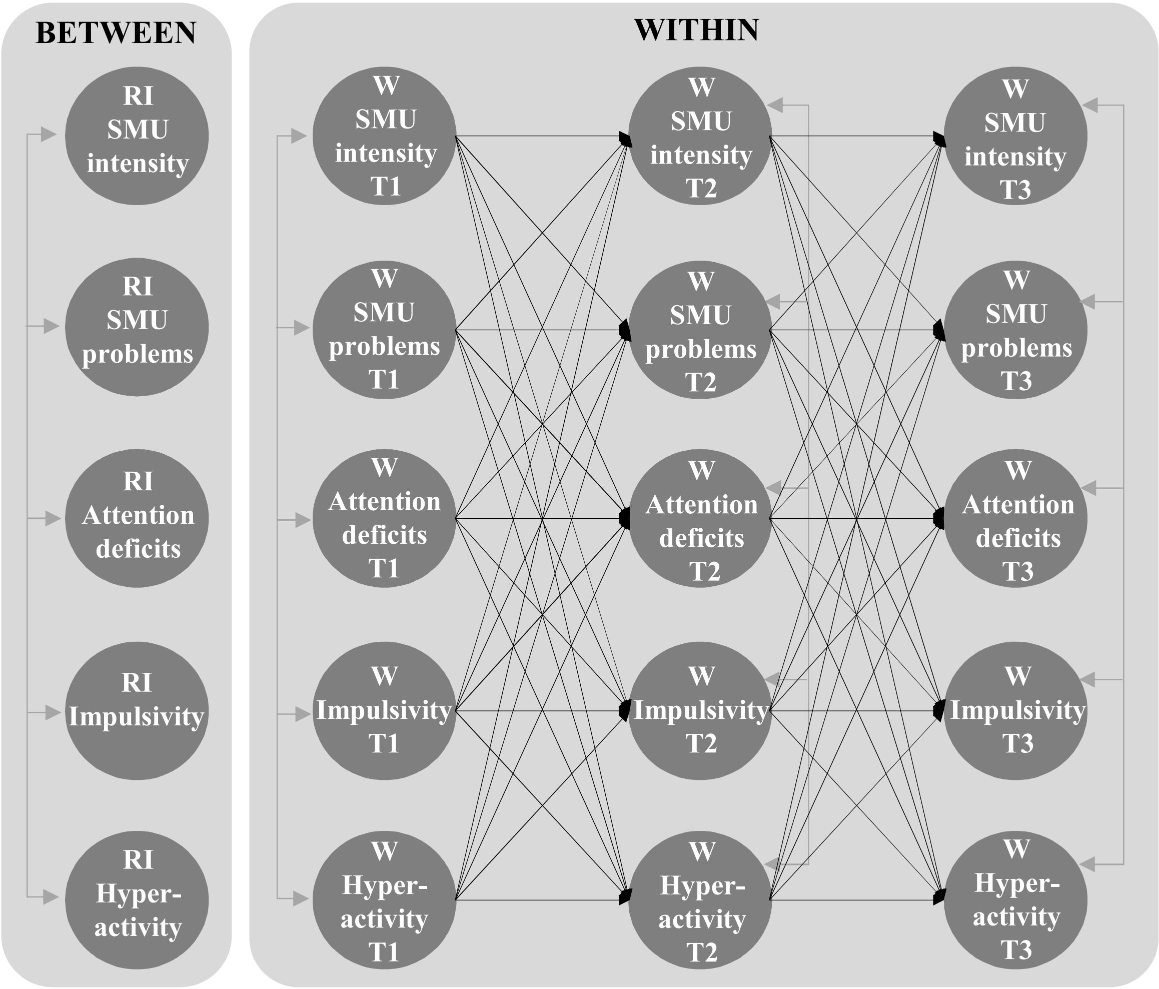

Figure S1. *Simplified illustration of the five-variable RI-CLPM.*

Circles in the between-area represent random intercepts (RI), which were extracted from their three respective computed factor scores (not shown in figure). Circles in the within-area represent within-person values (W), which were extracted from their respective computed factor score (not shown in figure). Black arrows represent cross-lagged and auto-regressive relations. All possible cross-lagged relations were specified for control purposes (e.g. attention deficits at T1 on impulsivity at T2). Light grey arrows represent (residual) correlations.
